# Reduction in host-finding behaviour in fungus-infected mosquitoes is correlated with reduction in olfactory receptor neuron responsiveness

**DOI:** 10.1186/1475-2875-10-219

**Published:** 2011-08-03

**Authors:** Justin George, Simon Blanford, Michael J Domingue, Matthew B Thomas, Andrew F Read, Thomas C Baker

**Affiliations:** 1 Department of Entomology, Pennsylvania State University, University Park, PA 16802, USA; 2 Center for Infectious Disease Dynamics, Pennsylvania State University, University Park, PA 16802, USA

## Abstract

**Background:**

Chemical insecticides against mosquitoes are a major component of malaria control worldwide. Fungal entomopathogens formulated as biopesticides and applied as insecticide residual sprays could augment current control strategies and mitigate the evolution of resistance to chemical-based insecticides.

**Methods:**

*Anopheles stephensi *mosquitoes were exposed to *Beauveria bassiana *or *Metarhizium acridum *fungal spores and sub-lethal effects of exposure to fungal infection were studied, especially the potential for reductions in feeding and host location behaviours related to olfaction. Electrophysiological techniques, such as electroantennogram, electropalpogram and single sensillum recording techniques were then employed to investigate how fungal exposure affected the olfactory responses in mosquitoes.

**Results:**

Exposure to *B. bassiana *caused significant mortality and reduced the propensity of mosquitoes to respond and fly to a feeding stimulus. Exposure to *M. acridum *spores induced a similar decline in feeding propensity, albeit more slowly than *B. bassiana *exposure. Reduced host-seeking responses following fungal exposure corresponded to reduced olfactory neuron responsiveness in both antennal electroantennogram and maxillary palp electropalpogram recordings. Single cell recordings from neurons on the palps confirmed that fungal-exposed behavioural non-responders exhibited significantly impaired responsiveness of neurons tuned specifically to 1-octen-3-ol and to a lesser degree, to CO_2_.

**Conclusions:**

Fungal infection reduces the responsiveness of mosquitoes to host odour cues, both behaviourally and neuronally. These pre-lethal effects are likely to synergize with fungal-induced mortality to further reduce the capacity of mosquito populations exposed to fungal biopesticides to transmit malaria.

## Background

Chemical insecticides targeting adult female mosquito vectors have been one of the most successful strategies employed for malaria control [1]. However, the effectiveness and sustainability of insecticide-based interventions, such as indoor residual sprays (IRS) and insecticide-treated nets (ITNs), is being undermined by evolution of insecticide resistance [1-8]. Accordingly, there is now a pressing need for novel control tools including alternative, non-chemical approaches [8]. One promising alternative is fungal entomopathogens formulated as biopesticides. A number of studies have demonstrated that residual contact with substrates treated with fungal sprays can lead to high levels of infection, reducing survival of a range of mosquito vector species, including insecticide resistant phenotypes [9-15]. Since it takes around two weeks for the malaria parasite to develop within a mosquito following a blood feed, the life-shortening effects of fungal infection can dramatically reduce malaria transmission potential [14-17]. In addition, fungal pathogens cause a range of pre- or sub-lethal effects in other arthropods including reductions in feeding [18-21], fecundity [22-26], flight performance [27,28] and predator avoidance [29,30], together with elevation of metabolic rate [31] and alteration in development [32]. For malaria control, reductions in feeding propensity could be particularly important since the parasite can only be transmitted during a blood feed and if feeding is reduced, it does not necessarily matter whether the mosquito is alive or not. To date, fungal infection has been shown to reduce feeding in *Anopheles*, *Culex *and *Aedes *mosquitoes [14,33,34]. The proximate mechanisms associated with such changes, however, remain unexplored.

Mosquitoes have a highly developed olfactory system that uses specialized olfactory receptor neurons (ORNs) to detect the odours emanating from their hosts [35]. They also use olfactory information to locate other food sources, mates, and oviposition sites. The peripheral olfactory organs that detect the olfactory cues are the antennae and maxillary palps. In *Aedes aegypti *and *Anopheles gambiae*, maxillary palps harbour a single morphological type of chemosensory sensillum, the capitate peg, which is innervated by three ORNs. One of these three ORNs is highly responsive to CO_2_. The second ORN is most sensitive to 1-octen-3-ol, which is a major component of human and other vertebrate volatiles [36,37]. In *An. gambiae*, this ORN expresses on its dendrites the odorant receptor (OR) called AgOR8, which is activated by 1-octen-3-ol [38]. The third ORN is tuned to a broad panel of odorants and expresses the OR named AgOR28 on its dendrite. Furthermore, *An. gambiae *responds with a significant dose-dependent electropalpogram (EPG) response profile to 1-octen-3-ol [37,38].

This study combines simple behavioural assays with electroantennogram (EAG) and electropalpogram (EPG) recordings to examine possible olfaction effects of fungal infection at the peripheral level. Single sensillum recordings (SSRs) were used to investigate individual capitate peg neuronal responses after fungal infection. Results show that in addition to survival, feeding propensity, upwind flight behaviour and the olfactory responses are all affected by fungal infection and depend on the fungal species examined. The results provide one of the first demonstrations of a pathogen having a functional impact on insect olfaction. This could have important implications for the effectiveness of fungi for vector control.

## Methods

### Mosquito rearing

*Anopheles stephensi *were reared under standard insectary conditions of 27°C, 80% humidity and 12:12 light: dark photo-period. Eggs were placed in plastic trays (25 cm × 25 cm × 7 cm) filled with 1.5 l of distilled water. To reduce variation in adult size at emergence, larvae were reared at a fixed density of 400 per tray. Larvae were fed Liquifry for five days and then on Tetrafin fish flakes. From approximately two weeks after egg hatch, pupae were collected daily and placed in emergence cages. The adults that emerged were fed *ad libitum *on a 10% glucose solution. All experiments used three to five day old adult female mosquitoes.

### Fungal isolates, formulation and application

Two species of entomopathogenic fungi were used that are known to vary in their impact on mosquitoes: *Beauveria bassiana *isolate IMI39150, which has been investigated in a number of previous publications and shown to have a marked impact on mosquito survival and performance [9,10,14,39], and *Metarhizium acridum *(formerly *Metarhizium anisopliae *var *acridum *[40] isolate IMI330189, which is a relatively specific pathogen of grasshoppers [41] but has been shown to infect mosquitoes although with only moderate virulence [39]. Both isolates were formulated separately in a mix of mineral oils (80% Isopar M:20% Ondina 22) and their concentrations adjusted to give 1 × 10^9 ^spores ml^-1^.

Application and mosquito exposure were as described by Bell *et al *[39]. In brief, spray applications of the spore formulation were applied to the insides of waxed cardboard cups using a hand-held artist's airbrush and allowed to dry. The spray method delivers an estimated 2 × 10^10 ^spores/m^2 ^of which approximately 2 × 10^8 ^spores/m^2 ^are actually deposited on the cup surface [39]. Mosquitoes were introduced to these cups for six hours resulting in a spore acquisition in the region of 2 × 10^4 ^spores/mosquito [39]. After exposure, mosquitoes were aspirated into mesh cages, provided with an *ad libitum *supply of 10% glucose, and housed in a controlled environment incubator at 26°C and 80% RH where they remained for the duration of the experiment. Control mosquitoes were handled in exactly the same way but were exposed to untreated cups. Each treatment had four replicate exposure cups per treatment with between 50 and 60 mosquitoes per replicate. After their time in the cups, mosquitoes were pooled into a single large cage according to their respective treatment, allowed to mix together and then redistributed into four replicate cages (~ 30 insects per cage) for survival assessment. Mortality was monitored daily and the trial stopped when all treated insects were dead or the trial had run for 14 days, whichever was sooner.

Three trials were run for feeding propensity and electrophysiology recordings. The first had three treatments: mosquitoes held in untreated cups ("UnExp"), or exposed to either *B. bassiana*-treated or *M. acridum-*treated cups ("Exp"). The second had two treatments: mosquitoes held in untreated cups or exposed to *B. bassiana*-treated cups. The third had un-exposed and *B. bassiana*-exposed insects and these were used for single sensillum recordings and wind tunnel flight assessments. In addition to the cages described above, a further cage (~ 120 insects) was set up for each treatment as a source of mosquitoes for SSR and flight trials.

### Propensity to feed and selection of mosquitoes for electrophysiological recording

*Beauveria bassiana*-exposed and unexposed mosquitoes were tested for their responsiveness to a feeding stimulus each day after exposure. *Metarhizium acridum*-exposed mosquitoes were tested every other day because previous trials had shown very little mortality in mosquitoes exposed to this fungal pathogen (unpublished data). The feeding stimulus comprised a 250 ml flask filled with hot tap water (water temperature 35-42°C) that was covered with one of the investigator's (SB) recently worn socks. This stimulus was placed within 0.5 cm of the side of the cage holding the mosquitoes. After one minute, the number of mosquitoes that landed on the mesh cover and began probing with their mouthparts was counted; these individuals are referred to as 'responders'.

Immediately after this count was made, a set of responding mosquitoes that had not been exposed to spores was removed from their cages and designated Unexposed Responders ("UnExpR"). Responding mosquitoes that had been exposed to spores were likewise removed from their cages and designated Exposed Responders ("ExpR). Mosquitoes that had not responded to the feeding-related stimulus were also selected and designated as Unexposed Non-Responders ("UnExpNR") and Exposed Non-Responders ("ExpNR"), respectively. Mosquitoes were taken evenly from across the four replicate cages within each treatment. Mosquitoes from each of the four groups were pooled into separate cardboard cups with mesh lids and used immediately for electrophysiological readings.

### Electrophysiology: EAGs, EPGs & SSR

To make a stable preparation for physiological recordings, methods similar to those established for mosquito antennal single neuron recordings were employed [42]. A female mosquito was anaesthetized at -20°C, for 75-90 s and mounted on a microscope slide between two pieces of double-sided tape. The insect was secured in place by covering half the thorax and abdomen using tape. Its antennae or palps were fixed against the lower surface using a piece of double-sided tape stuck to a cover slip resting on a small ball of dental wax, all of which helped facilitate manipulation of the mosquito in order to fix the antennae onto the tape. The cover slip was positioned parallel to the microscope slide at right angles to the mosquito head. Once mounted, the mosquito was placed in the electrophysiological recording rig under a Nikon Optihot microscope, which provided a highly magnified (×750) view of the antennal or maxillary palp sensilla. Two tungsten microelectrodes sharpened electrolytically in 10% KNO_2 _at 5-10 V to a 1 μm tip diameter were used for the electrophysiological recordings. One of these, the reference electrode, was inserted into the eye or thorax of the mosquito to form an electrical ground [43], using a Leitz mechanical micromanipulator. The second, recording electrode was connected to the preamplifier (10 ×, Syntech, Germany) and inserted into the antennae or palps using a Narishige hydraulic micromanipulator to complete the electrical circuit and to extracellularly record the summed DC potentials of olfactory receptor neurons on the antennae (EAGs) or palps (EPGs). EAGs and EPGs were performed on the fungus-exposed or un-exposed mosquitoes from 0-8 days after inoculation by inserting a tungsten electrode into the mosquito's antennae or palps [44]. For the *M. acridum *strain, EAGs were performed up to 13 days after inoculation. The preamplifier was connected to an analog-to-digital signal converter (IDAC, Syntech), which was connected to a computer for signal recording and visualization.

Single sensillum recordings were also done similarly by inserting the sharpened tungsten reference electrode into the eye and a tungsten recording electrode positioned into the base of the capitate peg sensillum on mosquito palps until electrical contact with the sensillum was established. Action potentials of the ORNs housed in the sensillum were amplified using a USB-IDAC interface amplifier (Syntech, Germany). The entire SSR recording was made only from capitate peg sensilla on mosquito palps, because this type of sensillum houses three ORNs, one that is tuned to CO_2_, another to 1-octen-3-ol [38] and third to other compounds that we did not assay. The neuronal responses of mosquitoes to 1-octen-3-ol and CO_2 _after exposure to fungal spores were determined by counting the number of spikes present during a 0.5 s pre-stimulus period and subtracting this background frequency from the frequency elicited during a 0.5 s post-stimulus delivery period.

### Odorant stimulus delivery

The 1-octen-3-ol was dissolved in hexane at a concentration of 1 μg/μl. A 10 μl aliquot of this solution was dispensed onto a filter paper, which was then inserted into a Pasteur pipette to create an odour cartridge having 10 μg of this stimulus. A constant airflow of charcoal-purified, humidified air was passed across the antennae through a 10 mm i.d. glass tube during the experiments. The odorant was delivered into this constant air stream via the Pasteur pipette whose tip was inserted through a small hole in the glass tube, 11 cm away from its end. A stimulus flow controller (Syntech, Hilversum, Netherlands) pulsed a 40 ml/s air stream through the cartridge for 0.05 seconds, effectively delivering 2 ml of volatiles from the odour cartridge into the air stream and onto the antenna. Either DC (EAG and EPG) or AC (single-sensillum) responses to the stimulus were recorded and analysed using Syntech Autospike software (Hilversum, Netherlands). The CO_2 _stimuli were delivered from a certified CO_2 _cylinder at the rate of 200 ml/min for 0.05 seconds at a room temperature of 26°C. In single-sensillum recordings, the ORN responding to CO_2 _exhibited large spikes that were easily differentiated from the smaller-spiking ORN responsive to 1-octen-3-ol. Spikes were counted for each of these ORNs in the single-sensillum recordings for all stimuli.

### Behavioural trials in wind tunnel

In order to further study the effect of fungal infection on mosquito flight and host-finding behaviour, we performed upwind flight trials in a wind tunnel using *B. bassiana*-exposed and unexposed mosquitoes from the same pools of mosquitoes that were used for SSR recordings. Mosquitoes were released into the heat-plus-odour plume in the tunnel and given a chance to fly upwind on days 2-5 after fungal exposure. The source of the plume was a 500 ml Erhlenmeyer flask filled with hot tap water (water temperature in the range of 45-50°C) and covered with one of the investigator's (JG) two-day-worn socks. The heat from the hot water provided heat cues and the worn socks provided the human volatile cues. Individual mosquitoes were placed, via a plastic transfer cup, into a 1.8 m × 0.6 m × 0.6 m acrylic glass wind tunnel, 1 m downwind of the feeding stimulus described above. The water in the flask was replaced every twenty minutes to ensure a constant temperature. Conditions in the tunnel were kept at 630 Lux light intensity at floor level 0.3 m/s wind velocity (activated charcoal filtered), 26-30°C and 50-75% RH. Ten mosquitoes were tested three times each and their upwind flight responses towards the stimulus source were recorded.

### Statistical analysis

Survival was analysed with Kaplan-Meier Survival analysis in SPSS for Mac v. 18. Median lethal times were calculated (± 95% confidence interval) and differences between treatments estimated using a Log Rank Test. For the behavioural wind tunnel trials, a logit link function was fit to the data using PROC GENMOD in SAS. The model assumed a binomial distribution for upwind flight tendency. This model allowed analysis of Day post-exposure and Treatment as factors influencing the proportion of successful flights. The Day*Treatment interaction effect was computed, along with Wald chi-square tests to test differences between treatments within each day (α = 0.05). Because three trials were run per individual, the REPEATED statement was used with an exchangeable within-subject correlation matrix. EAG responses were square-root transformed to normalize the data and remove variance heterogeneity among treatments. Transformed data were analysed using analysis of variance (SAS v. 9.2). The factors considered were Day post-exposure when the measurements were made, and fungal and behavioural treatment grouping. The treatments included the responding and non-responding behaviours in the spore-exposed or unexposed groups: ExpR; ExpNR; UnExpR; and UnExpNR. The Day*Treatment interaction was also included in the model. Comparisons among the four treatments were made using the Tukey-Kramer adjustment (α = 0.05). Single-sensillum recordings were similarly analysed using ANOVA, with some minor differences. For these analyses no transformation of the data was necessary. There were only two treatment groups with respect to fungal infection, with no behavioural analyses having been performed. Also, because more replicates were performed at fewer days, it was possible to perform comparisons among the different Day*Treatment combinations. Finally, because 1-octen-3-ol was always presented first in our recordings, whereas the order of presentation of CO_2 _versus the blend of 1-octen-3-ol plus CO_2 _was varied, the effect of this difference in the order of presentation was included as a random factor when appropriate.

## Results

### Mosquito survival and response to feeding-related stimuli

**Figure 1:**
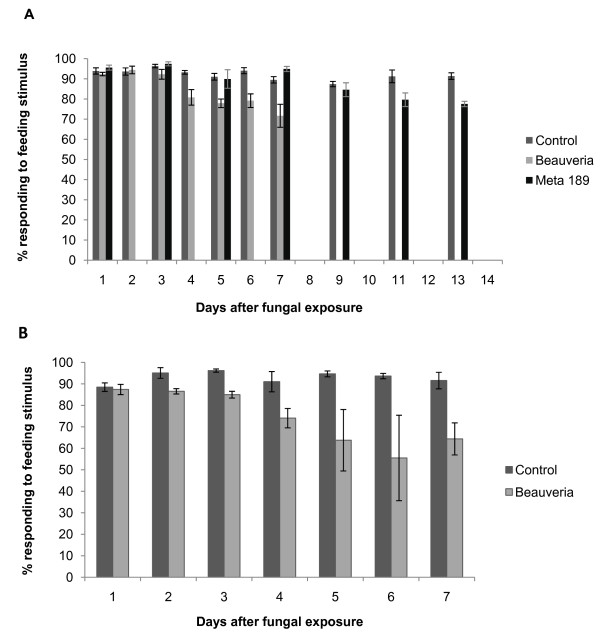
**The responses of *An. stephensi *female mosquitoes to a feeding-related heat-plus-odour stimulus**. Behaviour was assessed each day following exposure to *B. bassiana *or *M. acridum *fungal spores. "Controls" received no spores. **A) **Mosquitoes from Trial 1 in which individuals were drawn to assess EAG olfactory responses from antennae. **B) **Mosquitoes from Trial 2, drawn to assess olfactory EPG responses from palps. Median day of mortality for the *B. bassiana *groups was Day 6, and so few survivors were available for the behavioural assay after Day 7, in contrast to the *M. acridum *and Control groups that had low mortality (see Results text). Responsiveness of *M. acridum*-exposed mosquitoes was assayed every second day. Brackets around the means denote ± 1 SEM.

Mosquitoes that had been exposed to *B. bassiana *spores died more quickly than unexposed mosquitoes or those exposed to *M. acridum *spores. In trial 1, exposure to *B. bassiana *was associated with a steady increase in mortality, with 100% of the exposed mosquitoes dead by day 13 and median lethal time for this group of 6.0 (5.81-6.19) days. The median lethal times for *M. acridum*-exposed mosquitoes and the unexposed control group were 11.0 (10.21-11.79) and 10.0 (8.97-11.03) days, respectively (Log Rank (Mantel Cox) statistic (LR stat) = 0.37, *P *= 0.54). Of the surviving mosquitoes, those that had been exposed to *B. bassiana *exhibited reduced responsiveness to host-related feeding cues from day 4 onwards, relative to control and to *M. acridum-*exposed mosquitoes (Figure 1A). Additionally, even though mortality was negligible following exposure to *M. acridum*, these mosquitoes exhibited reduced responsiveness to host-related feeding cues relative to the control group from day 11 (Figure 1A).

In trial 2, *Beauveria*-exposed mosquitoes again showed a higher daily mortality than control insects, with a median lethal time of 6.0 (5.79-6.21) days (LR stat = 291.8, *P *< 0.001) (no median lethal time could be computed for the control group because survival was 72% when the assay was censored on day 10). As in trial 1, surviving *B. bassiana*-exposed mosquitoes exhibited reduced responsiveness to feeding cues relative to unexposed controls with clear differences apparent by day 4 and continuing to the end of the trial (Figure 1B).

**Figure 2:**
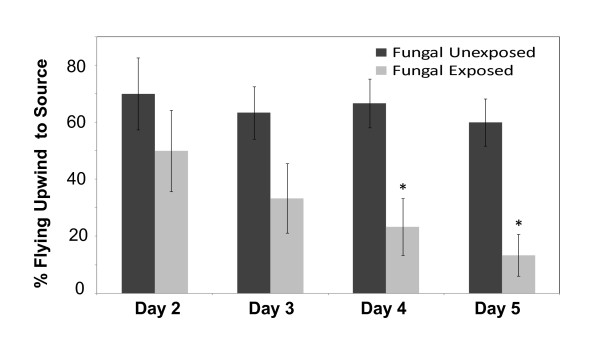
**Flight tunnel behavioural assay**. *An. stephensi *in-flight host-finding ability was assessed in the days following exposure to *B. bassiana *spores. On each day, ten mosquitoes from each of the fungal-exposed or unexposed groups were assessed three times for their responsiveness to the heat-plus-odour source. Mean percentage (+/- standard error) of mosquitoes flying upwind is shown. Asterisks denote fungal exposed groups having significantly lower upwind flight responses than the unexposed group within a given day using Wald chi-square tests (α = 0.05 after Bonferoni-correction).

The *B. bassiana*-exposed mosquitoes used in the single-cell recordings and wind tunnel flight trials also showed significant reduction in survival relative to controls (LR stat = 229.9, *P *< 0.001), with median lethal time of 6.0 (5.66 - 6.34) days. Control survival was > 80% by day 11 when the trials were terminated. These exposed mosquitoes showed reduced responsiveness to host-related cues. Beginning two days post-exposure, mosquitoes were significantly less likely to take off and fly 1 m upwind in the heat-plus-odour plume than the unexposed controls (Figure 2). The repeated measures logistic regression model indicates a significant effect of fungal *Infection *(chi-square = 14.96, d.f. = 1, p < 0.0001), but not of *Day *(chi-square = 4.38, d.f. = 3, p = 0.22), or the Day*Infection interaction (chi-square = 2.41, d.f. = 3, p = 0.49). The exchangeable working correlation is 0.085. Pairwise comparisons were made among the Day*Infections combinations.

### EAG and EPG responsiveness in fungal-exposed and unexposed mosquitoes

**Figure 3:**
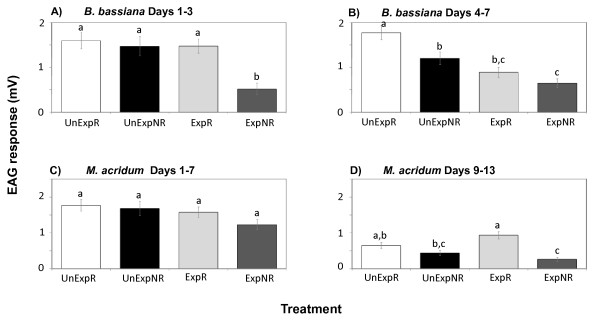
**Mean EAG amplitudes (± SE) from female *An. stephensi *antennae**. EAGs were in response to 1-octen-3-ol from both behaviourally responsive and non-responsive mosquitoes that had or had not been exposed to *B. bassiana *or *M. acridum *spores. **UnExp **= unexposed to fungus, **Exp **= exposed to fungus. **R **= behavioural responders in feeding stimulus assay, **NR **= behavioural non-responders in feeding stimulus assay. Data are grouped by days according to the earlier and later time-periods in which behavioural changes in responsiveness began to occur (See Figure 1): **A) ***B. bassiana *exposure experiment, in which recordings were made from 1 to 3 days after fungus exposure, **B) ***B. bassiana *exposure experiment, in which recordings were made from 4 to 7 days after fungus exposure, **C) ***M. acridum *exposure experiment, in which recordings were made from 1 to 7 days after fungus exposure, **D) ***M. acridum *exposure experiment, in which recordings were made from 9 to 13 days after fungus exposure Within each grouping, treatments having no letters in common are significantly different (Tukey-Kramer adjustment, α = 0.05).

During the first three days post-exposure, when behavioural responsiveness was apparently little affected by fungal exposure (Figure 1A), there were differences in EAG amplitudes among the treatments (p = 0.0001, Table 1), with *B. bassiana*-exposed behaviourally non-responsive mosquitoes, ExpNR, exhibiting significantly lower EAG responses to 1-octen-3-ol than all other treatments (Figure 3A). As the fungal infection progressed (days 4-7), and behavioural responsiveness decreased (Figure 1A), there continued to be differences in the EAGs among treatments (p < 0.0001, Table 1). The EAGs of ExpNR mosquitoes remained significantly lower than the similarly behaviourally unresponsive unexposed mosquitoes, UnExpNR (Figure 3B). Furthermore, the fungus-exposed responders, ExpR, also exhibited lower EAGs compared to unexposed responding mosquitoes (Figure 3B).

**Table 1:** Summary of ANOVA performed in the physiological experiments, using electroantenogram (EAG), electropalpogram (EPG), or single-sensillum recording (SSR).

Treatment	Compound	Recording	Fungus	Day	Day*Exposure	Order
B. bassiana Day 1-3	1-octen-3-ol	EAG	F = 9.34; 3,34 d.f. p = 0.0001	F = 15.8; 2,34 d.f. p < 0.0001	F = 1.92; 6,34 d.f. p = 0.11	na
*B. bassiana* Day 4-7	1-octen-3-ol	EAG	F = 14.8; 3,60 d.f. p < 0.0001	F = 1.10; 3,60 d.f. p = 0.36	F = 2.33; 9,60 d.f. p = 0.0025	na
*M. acridum* Day 1-7	1-octen-3-ol	EAG	F = 2.32; 3,71 d.f. p = 0.085	F = 14.5; 3,71 d.f. p < 0.0001	F = 3.33; 9,71 d.f. p = 0.0025	na
*M. acridum* Day 9-13	1-octen-3-ol	EAG	F = 13.6; 3,59 d.f. p < 0.0001	F = 111; 2,59 d.f. p < 0.0001	F = 4.41; 6,59 d.f. p = 0.0013	na

*B. bassiana* Day 1-3	1-octen-3-ol	EPG	F = 1.51; 3,45 d.f. p < 0.22	F = 6.14; 2,45 d.f. p = 0.0044	F = 0.85; 6,45 d.f. p = 0.54	na
*B. bassiana* Day 4-7	1-octen-3-ol	EPG	F = 19.6; 3,50 d.f. p < 0.0001),	F = 20.6; 3,50 d.f. p < 0.0001	F = 4.51; 9,50 d.f. p = 0.0002	na
*B. bassiana* Day 1-3	CO_2_	EPG	F = 1.15; 3,45 d.f. p = 0.34, Fig. 4C	F = 6.24; 2,45 d.f. p = 0.0041),	F = 1.26; 6,45 d.f. p = 0.29	na
*B. bassiana* Day 4-7	CO_2_	EPG	F = 11.1; 3,50 d.f. p < 0.0001).	F = 28.9; 3,50 d.f. p < 0.0001)	F = 6.79; 9,50 d.f. p < 0.0001).	na
*B. bassiana* Day 1-3	1-octen-3-ol & CO_2_	EPG	F = 2.81; 3,45 d.f. p = 0.05	F = 15.8; 2,45 d.f. p < 0.0001	F = 1.09; 6,45 d.f. p = 0.38	na
*B. bassiana* Day 4-7	1-octen-3-ol & CO_2_	EPG	F = 13.4; 3,50 d.f. p < 0.0001	F = 18.5; 3,50 d.f. p < 0.0001	F = 3.26; 9,50 d.f. p = 0.0034	na

*B. bassiana*	1-octen-3-ol	SSR	F = 53.3; 1,28 d.f. p < 0.0001	F = 11.9; 2,28 d.f. p = 0.0002	F = 19.6; 2,28 d.f. p < 0.0001	na
*B. bassiana*	CO_2_	SSR	F = 0.03; 1,27 d.f. p = 0.86	F = 16.1; 2,27 d.f. p < 0.0001	F = 7.56; 2,27 d.f. p = 0.0025	F = 2.08; 1,27 d.f. p = 0.16
*B. bassiana*	1-octen-3-ol (in blend)	SSR	F = 10.5; 1,27 d.f. p = 0.0032	F = 7.35; 2,27 d.f. p = 0.0028	F = 1.72; 2,27 d.f. p = 0.20	F = 1.00; 1,27 d.f. p = 0.33
*B. bassiana*	CO_2_ (in blend)	SSR	F = 1.14; 1,27 d.f. p = 0.29	F = 11.8; 2,27 d.f. p = 0.0002	F = 0.23; 2,27 d.f. p = 0.80	F = 3.70; 1,27 d.f. p = 0.065

Control or fungus-exposed mosquitoes were exposed to either 1-ocen-3-ol, CO_2_, or a blend of these compounds while performing these techniques as described in the text.

Reduced EAG amplitudes in response to 1-octen-3-ol were not seen in mosquitoes exposed to *M. acridum *over the first seven days post-infection (p = 0.085, Table 1, Figure 3C). For day 9-13 there were significant differences among the infection and behaviourally classified treatment means (p < 0.0001, Table 1). On these later days, the EAG amplitudes of *M. acridum*-exposed non-responders, ExpNR, were lower than those of the similarly fungus-exposed, but responsive, ExpR, mosquitoes (Figure 3D).

**Figure 4:**
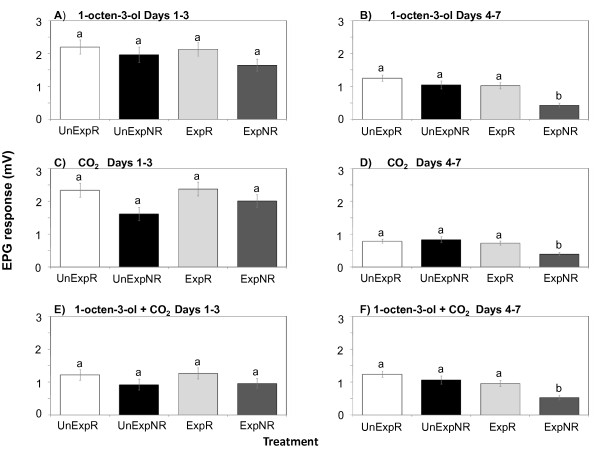
**Mean EPG amplitudes (± SE) from female *An. stephensi *maxillary palps**. EPGs were in response to 1-octen-3-ol and carbon dioxide from both behaviourally responsive and non-responsive mosquitoes that had or had not been exposed to *B. bassiana *spores. **UnExp **= unexposed to fungus, **Exp **= exposed to fungus. **R **= behavioural responders in feeding stimulus assay, **NR **= behavioural non-responders in feeding stimulus assay. Data are grouped by days according to the earlier and later time-periods in which strong behavioural differences in responsiveness occurred (See Figure 1): **A) **1-octen-3-ol response, in which recordings were made from 1 to 3 days after fungus exposure; **B) **1-octen-3-ol response, in which recordings were made from 4 to 7 days after fungus exposure; **C) **carbon dioxide response, in which recordings were made from 1 to 3 days after fungus exposure; **D) **carbon dioxide response, in which recordings were made from 4 to 7 days after fungus exposure; **E) **1-octen-3-ol plus carbon dioxide response, in which recordings were made from 1 to 3 days after fungus exposure; **F) **1-octen-3-ol plus carbon dioxide response, in which recordings were made from 4 to 7 days after fungus exposure. Within each grouping, treatments having no letters in common are significantly different (Tukey-Kramer adjustment, α = 0.05).

For EPG recordings, there were no significant differences in response to 1-octen-3-ol among treatment in the group measured within three days of exposure (p = 0.22, Table 1, Figure 4A). However, significant differences among the treatments arose in the 4-7 day period (p < 0.0001, Table 1), with *B. bassiana*-ExpNR mosquitoes having significantly lower EPGs than UnExpNR mosquitoes (Figure 4B). Thus the reduction in EPG response occurs 4 days post-exposure, which was when we began to see a decline in the behavioural responsiveness of fungus-exposed mosquitoes in the feeding-related stimulus trials (Figure 1B). There was no significant difference in the EPG responses of mosquitoes in the earlier stages of exposure, days 1-3 (Figure 4A). Thus, reduced EPG response to odorants in fungus-infected mosquitoes manifested itself in later stages of infection, days 4-7 post-exposure. As in the EAG recordings, although there was a reduced overall activity level for all non-responders during this period, there was an additional significant reduction imposed on the palps' olfactory system by fungal infection (Figure 4B).

Similar to the 1-octen-3-ol response pattern, in the early stages of infection exposure (days 1-3), there were no differences between EPG amplitudes in response to CO_2 _amongst the different mosquito classes (p = 0.34, Table 1, Figure 4C). However, at the later stages of infection (days 4-7), there were significant differences between EPG amplitudes in response to CO_2 _amongst the different mosquito classes (p < 0.0001, Table 1). More specifically, ExpNR mosquitoes exhibited significantly reduced EPG responses to CO_2 _compared to both UnExpNR and ExpR mosquitoes in later stages of infection, days 4-7 (Figure 4D).

There was only a marginal indication that mosquito classes differed in EPG responses to the mixture of 1-octen-3-ol and CO_2 _in day 1-3 (p = 0.05, Table 1), with no specific comparisons among treatment means showing significant differences (Figure 4E). In response to the mixture of 1-octen-3-ol and CO_2 _in days 4-7, there was a significant difference among treatments (p < 0.0001, Table 1), with the UnExpNR group again having a significantly weaker response than the other groups (Figure 4F). Thus, the EPG responses to CO_2 _and 1-octen-3-ol puffed as a mixture (Figures 4E, F) were similar to those in which 1-octen-3-ol or CO_2 _were puffed alone (Figures 4A-D).

### Single-sensillum recordings

**Figure 5:**
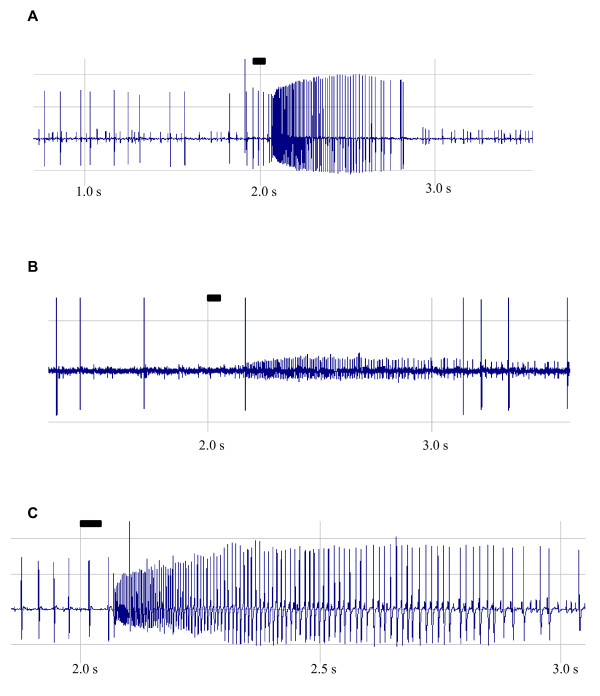
**Sample recordings of action potentials from ORNs in the capitate peg sensillum of female *An. stephensi***. **A) **The large-spiking ORN firing in response to CO_2_. **B) **The smaller-spiking ORN firing in response to 10 μg 1-octen-3-ol. **C) **The smaller and larger spiking ORNs firing in response to CO2+ 1-octen-3-ol blend (10 μg). Rectangular bar shows stimulus duration of 0.05 s.

Good single-cell recordings were obtained from the capitate peg sensilla of the maxillary palps (Figure 5). In all the preparations, a large-spiking ORN was clearly responsive to CO_2 _(Figure 5A), whereas a much smaller-spiking ORN in this same sensillum was most responsive to 1-octen-3-ol (Figure 5B). The combined spike response to CO_2 _and 1-octen-3-ol blend is shown as Figure 5C.

**Figure 6:**
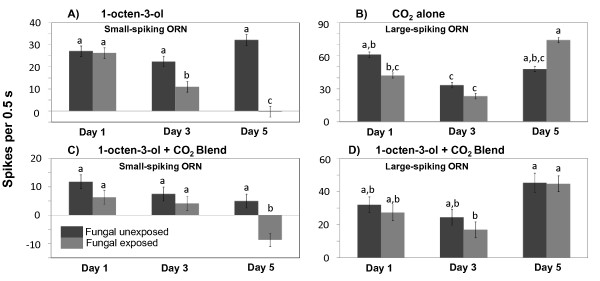
**Single cell action potential frequencies from ORNs in the capitate peg sensillum of female *An. stephensi *maxillary palps**. **A) **Response of the small spiking octenol-tuned ORN to 1-octen-3-ol (10 μg); **B) **response of the larger spiking CO_2_-tuned ORN to CO_2 _; **C) **response of the small-spiking octenol-tuned ORN to a blend of 1-octen-3-ol (10 μg) and CO_2 _; **D) **response of the large-spiking CO_2_-tuned ORN to a blend of 1-octen-3-ol (10 μg) and CO_2_. Spike frequency was assessed during 0.5 s post-stimulus delivery and background spike frequency during the pre-stimulus period was subtracted from the post-stimulus frequency (see Methods text). The bars on the means represent standard errors. In nearly all cases there were n = 6 replicates per odorant per day. Treatments within each of these groups having no letters in common are significantly different at α = 0.05 (Tukey-Kramer adjustment).

Responses by the ORN in capitate peg sensilla tuned to 1-octen-3-ol, when presented with that compound alone, exhibited significantly diminished spike frequencies on Days 3 and 5 following fungal exposure (Figure 6A, p < 0.05; Table 1) compared to the frequencies of this ORN in unexposed control-group mosquitoes. There were strongly significant effects of the number of days after infection (p = 0.0002, Table 1) and the Day*Infection interaction (p < 0.0001, Table 1). Responses of this ORN in fungus-exposed mosquitoes on Day 1 post-exposure compared to those of the unexposed group were not significantly different (Figure 6A). Spike frequencies of the 1-octen-3-ol ORN were generally lower when exposed to a blend of 1-octen-3-ol plus CO_2 _(Figure 6C) than when exposed to CO_2 _alone (Figure 6A), but by Day 5 they were significantly lower in fungus-exposed mosquitoes than those of the unexposed control group (Figure 6C, p = 0.05, Table 1).

A similar pattern of diminution of response of the large-spiking ORN tuned to CO_2 _after fungus exposure was not apparent, whether CO_2 _was puffed alone (p = 0.86, Table 1, Figure 6B) or as part of a blend along with 1-octen-3-ol (p = 0.29, Table 1, Figure 6D).

## Discussion

This study confirmed numerous previous studies indicating this particular isolate of *B. bassiana *to be lethal to mosquitoes [10,14,33]. Transferred to the field, the significant life shortening observed from even the relatively modest doses delivered in the current trials system should result in reduced vectorial capacity. However, the findings from this research supports previous studies [14,34], that surviving fungal exposed mosquitoes exhibit reduced propensity to feed, which would reduce malaria transmission still further. Notably, the changed feeding behaviour (including reductions in upwind flight towards host cues) appears linked, at least in part, to reductions in responsiveness to host-related feeding stimuli.

As expected, the *M. acridum *isolate had a much less marked effect on mosquito survival and based on simple mortality assessments, might lead to the conclusion that exposed mosquitoes remain uninfected by this relatively acridid-specific pathogen. However, the behavioural and electrophysiological measurements indicate that mosquitoes suffered sub-lethal effects from *M. acridum *infection resulting in reduced feeding propensity and impaired olfaction. Many species of mosquitoes, including the major malaria vector *An. gambiae*, utilize CO_2 _and 1-octen-3-ol as olfactory cues in host-seeking behaviours [38]. CO_2 _stimulation synergizes responses to host odour and by itself induces take-off and sustained flight behaviours in host-seeking anophelines [45,46]. Some mosquito species use the discontinuous pulses from breath-emitted CO_2 _to fly upwind during their host-seeking behaviour, whereas other animal odorants, such as lactic acid, can contribute to additional host-locating upwind flight without being pulsed [47]. Reception of CO_2 _integrates these odorant inputs to drive sustained upwind flight [35,38]. The EAG, EPG, and single-cell neurophysiological recordings from behaviourally unresponsive fungus-exposed mosquitoes all show a corresponding reduction in neuron responsiveness to the host-related volatile compound, 1-octen-3-ol. These diminished neuronal responses were significantly lower than those obtained from behaviourally unresponsive control mosquitoes, indicating that the olfactory capabilities of the fungal-exposed mosquitoes are impaired and contribute to their behavioural reduction to feeding cues.

Why olfactory responsiveness to 1-octen-3-ol was so clearly and repeatedly reduced by *B. bassiana *exposure and that of CO_2 _is unknown. Because the maxillary palp ORNs responsive to these compounds co-reside in the same capitate peg type of sensillum, it seems unlikely that there is some kind of physical or chemical impairment producing blockage of cuticular pores reducing entry of molecules from the outside, or some kind of general ion imbalance in the receptor lymph caused by fungal infection. However, it should be noted that the odorant receptor type that is expressed on the ORN tuned to 1-octen-3-ol is a true odorant receptor (OR), whereas the "OR" that is expressed on the ORN tuned to CO_2 _is a gustatory receptor (GR) [48,49]. ORs are coupled to an extra protein, *Orco*, [50] which forms a heterodimer with the odorant-ligand-accepting second receptor protein on the dendritic membrane. GRs are not coupled to *Orco *[49]. In the case of the ORN tuned to 1-octen-3-ol, in *An. gambiae *this co-receptor that is tuned to 1-octen-3-ol is named AgOR8 [38,51]. These differences in the dendritic membrane-bound protein receptors on these two types of neurons may possibly be related to the degree of fungal infection-related reduction of responsiveness in the ORN tuned to 1-octen-3-ol compared to that of the ORN tuned to CO_2_.

Fungal pathogens are known to release a range of toxins and secondary metabolites during infection that compete for energy reserves and cause general disruption of host tissues [52-54]. Studies on the pre-lethal effects of fungal infection in locusts demonstrate that infected locusts feed less and are less able to sustain flight compared with uninfected controls [28,29], with "fever" responses [55] and fungus-secreted "energy scavenging" enzymes suggested to play a role [56]. The results presented here add a new dimension to such pre or sub-lethal effects, indicating an impact of fungal infection on insect sensory capability.

## Conclusions

This is the first study that, to our knowledge, correlates fungal-induced reductions in mosquito host-seeking behaviour with a decline in electrophysiological sensitivity of the olfactory apparatus. In the context of malaria control, reductions in response to feeding-related cues and upwind flight behaviour could add considerably to the direct impacts of fungal infection on mosquito survival. The importance of such subtle pre- or sub-lethal effects of fungal infection can only be tested fully under more realistic semi-field or field conditions. Placed in a more complicated environmental context with diverse and variable cues, it is possible that the effects we identified here might be relatively unimportant. Alternatively, and perhaps more likely, given we have been working over small spatial scales and dealing with young, healthy insects maintained under ideal laboratory conditions, the behavioural, physiological and survival effects we have found so far are *underestimates *of the potential of fungi to reduce malaria transmission.

## Competing interests

The authors declare that they have no competing interests.

## Authors' contributions

The initial experimental designs were developed by JG, SB, MBT, AFR and TCB. JG carried out the behavioral and electrophysiological experiments and drafted the manuscript. SB involved in behavioral studies, design and coordination of experiments. MJD performed the statistical analysis. SB, MJD, MBT, AFD and TCB contributed to drafting the final manuscript. All authors read and approved the final manuscript.
